# Common Genetic Variants in *TRIO* Are Associated With Autism in Chinese Han Population

**DOI:** 10.1155/genr/7762302

**Published:** 2025-12-17

**Authors:** Han Shen, Xiaoxuan Sun, Ziqi Wang, Miaomiao Jiang, Jinxin Wang, Tianlan Lu, Weihua Yue, Dai Zhang, Lifang Wang, Jun Li

**Affiliations:** ^1^ Peking University Sixth Hospital, NHC Key Laboratory of Mental Health (Peking University), National Clinical Research Center for Mental Disorders (Peking University Sixth Hospital), Peking University Institute of Mental Health, Key Laboratory of Mental Health, Chinese Academy of Medical Sciences, Beijing, China, cams.ac.cn; ^2^ Beijing Anding Hospital, Capital Medical University, Beijing, China, ccmu.edu.cn; ^3^ State Key Laboratory of Cognitive Neuroscience and Learning & IDG/McGovern Institute for Brain Research, Beijing Normal University, Beijing, China, bnu.edu.cn; ^4^ PKU-IDG/McGovern Institute for Brain Research, Peking University, Beijing, China, pku.edu.cn; ^5^ Institute for Brain Research and Rehabilitation (IBRR), South China Normal University, Guangzhou, China, scnu.edu.cn; ^6^ Changping Laboratory, Beijing, China

**Keywords:** autism spectrum disorders (ASDs)_1_, family-based association study_2_, genetic association_5_, single-nucleotide polymorphisms (SNPs)_3_, TRIO_4_

## Abstract

Autism spectrum disorders (ASDs) are a group of neurodevelopmental disorders with high heritability. Nevertheless, the involvement of genetic variants in ASDs is not fully understood. One gene of interest is *TRIO*, which encodes a large protein that aids in GDP‐to‐GTP exchange as a Ras homologous (Rho) guanine nucleotide exchange factor (GEF), facilitating cytoskeleton reorganization. Thus, it plays crucial roles in neuronal migration, neurite outgrowth, and synaptic transmission. *De novo* mutations in *TRIO* have been extensively reported in the pathogenesis of ASDs. However, no evidence currently supports the genetic association between common variants in *TRIO* and ASDs. To investigate the role of common genetic variations in autism risk, we analyzed 12 tagging single‐nucleotide polymorphisms (SNPs) in the *TRIO* gene. These tagging SNPs captured an average of 75% of all common variations in *TRIO* with a minor allele frequency (MAF) > 5%. Using the family‐based association study in 239 Chinese Han autism trios, we identified the significant association of three SNPs (rs32593, rs33005, and rs27479) with autism. To confirm the association, the sample size was expanded to 427 trios by recruiting 188 additional trios. Our findings across all 427 trios confirmed that A allele of rs32593, G allele of rs33005, and C allele of rs27479 showed a preferential transmission to the affected offspring (rs32593: A > G, Z = 2.600, *p* = 0.0093; rs33005: G > T, Z = 2.978, *p* = 0.0029; rs27479: C > A, Z = 3.214, *p* = 0.0013) after Bonferroni’s correction (*p* < 0.0042). Haplotype analyses showed that one haplotype (A‐G) constructed from rs32593 and rs33005 was significantly associated with autism (*p* = 0.0064; Global *p* = 0.022). These results suggested that the common variants in *TRIO* might be involved in the susceptibility to autism in the Chinese Han population.

## 1. Introduction

Autism spectrum disorders (ASDs) are a spectrum of heterogeneous neurodevelopmental disorders characterized by social interaction and communication deficits as well as limited, repetitive activities and interests [1–3]. The prevalence of ASDs is gradually increasing year by year. According to the Centers for Disease Control and Prevention’s (CDC) Morbidity and Mortality Weekly Report (MMWR), the overall prevalence of ASDs was 27.6 per 1000 (one in 36) children aged 8 years in the United States. An estimated prevalence of ASDs among 6‐ to 12‐year‐old children was 0.70% in China [2, 4]. Genetic analysis has been integral in uncovering the underlying causes of ASDs [2]. Twin studies indicated a high heritability rate of approximately 80%–90% for ASDs [1, 3, 5]. Recently, researchers have capitalized on advanced sequencing methods such as whole‐exome sequencing (WES) and whole‐genome sequencing (WGS) to examine the genetic factors contributing to ASDs across large cohorts [6, 7]. These studies shed light on the function of rare genetic variants, both *de novo* and inherited, in ASD pathogenesis [8–10]. However, the involvement of common variants, which are largely located in noncoding regions, in the etiology of autism remains unknown.

*TRIO* is a member of the Dbl‐homology guanine nucleotide exchange factor (DH‐GEF) subfamily, which plays a role in the signaling of Rho guanosine triphosphatases (GTPases) [11, 12]. These Rho GTPases (i.e., Rac, Cdc42, and RhoA) are crucial for various neurodevelopmental processes including neurogenesis, neuronal migration, and synaptic formation, by regulating the dynamics of the actin cytoskeleton [11, 13, 14]. In particular, two short isoforms of *Trio*, known as *Trio9* and *Trio8*, have high expression levels in the brain [15, 16]. Our recent studies demonstrated that *Trio* is critical for embryonic radial and tangential migration as a central regulator of actin dynamics by affecting distinct signaling pathways, which are involved in autism‐related behaviors [17, 18]. In addition, the roles of *TRIO* in promoting axon growth, dendritic spine formation, and synaptic function have also been extensively studied in vivo and in vitro [2].

The increased attention focused on *TRIO* stems from the growing evidence for its role in the etiology of neurodevelopmental disorders including ASDs [19–21]. Recent WES and WGS studies in ASDs identified an increased number of disease‐related rare *de novo* and inherited variations in *TRIO* [21–24]. However, there is no evidence for the genetic association between common variants in *TRIO* and ASDs. Here, we employed a family‐based association study in the Chinese Han population to highlight the common variants in *TRIO* that might increase disease susceptibility. We revealed that the single‐nucleotide polymorphisms (SNPs) rs32593, rs33005, and rs27479, as well as their haplotypes, are significantly associated with autism, indicating that the common variants in *TRIO* might also be involved in the susceptibility of autism.

## 2. Materials and Methods

### 2.1. Ethics Statement

This research was approved by the Ethics Committee of Peking University Sixth Hospital. All participants in this study were required to provide written informed consent, with parental or guardian consent obtained for children.

### 2.2. Participants

The participants were recruited from Peking University Sixth Hospital in Beijing, China. Initially, we recruited 239 autism trios with autistic patients and their biological parents of Chinese Han descent, including 226 boys and 13 girls among the affected children. These affected children were between the ages of 2 and 17, with an average age of 7.5 at the time of the clinical evaluation. We first identified three significantly associated SNPs among 12 candidate loci in 239 autism trios. Subsequently, we expanded our sample size up to 427 by adding an additional 188 trios. All newly recruited autistic children in this stage were male, with a median age of 6.0 years. Then, the association of these three SNPs with autism was further validated in a total of 427 autism trios. Table 1 summarizes the characteristics of all participants.

**Table 1:** Demographic characteristics of all participants.

	Family units	Age^a^	Sex (male/female)
Autistic children	427	7.0 (2.0, 17.0)	414/13
Biological father	427	34.00 (32.00, 37.00)	—
Biological mother	427	32.00 (30.00, 35.00)	—

^a^Age was statistically described using the median (lower quartile, upper quartile).

In this study, the diagnosis of autistic disorder was established by at least two skilled psychiatrists following the criteria outlined in the Diagnostic and Statistical Manual of Mental Disorders, Fourth Edition (DSM‐IV). The assessments were carried out using the Childhood Autism Rating Scale (CARS) and the Autism Behavior Checklist (ABC). To ensure homogeneity in the study population, only individuals diagnosed with autism were included, whereas those with conditions such as Asperger’s syndrome, Fragile X syndrome, chromosomal abnormalities, tuberous sclerosis, other neurological conditions, familial/inherited diseases, or severe mental disorders were excluded. The social and communicative abilities of the parents of autistic children were also evaluated, and none of them met the autism diagnostic criteria outlined in the DSM‐IV. DNA was extracted from peripheral blood samples of all individuals involved in the study, using Qiagen QIAamp DNA Kits, following informed consent procedures.

### 2.3. SNP Selection

**Figure 1:**
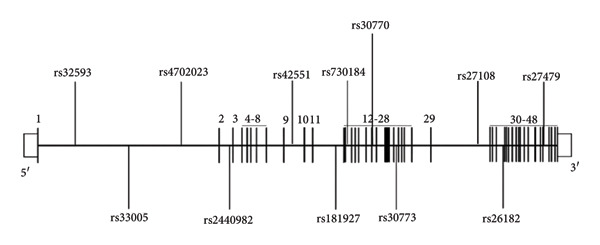
A diagram of the position of selected 12 tag SNPs in *TRIO*. A diagram of the structure of *TRIO*, with exons in black. The selected 12 tag SNPs were noted.

The HapMap genotype dataset (http://hapmap.ncbi.nlm.nih.gov/) was utilized to acquire the genotyping information for *TRIO* polymorphisms in the Chinese Han population in Beijing (CHB) from HapMap phases II and III. The SNPs examined in this study have a minor allele frequency (MAF) > 0.05 in the CHB population. Furthermore, the Tagger module included in the Haploview version 4.2 program was used for pairwise tagging to identify SNPs that could successfully capture the common genetic variations within each gene (*r* ^2^ > 0.8). In the current investigation, a total of 12 tag SNPs in *TRIO* were identified from 14,143,342 bp to 14,510,204 bp on Chromosome 5 with a mean range of 29 kb (GRCh38, National Center for Biotechnology Information [NCBI]) (Figure 1).

### 2.4. DNA Extraction and Genotyping

All participants provided peripheral blood samples, and genomic DNA was extracted using the Qiagen QIAamp DNA Mini Kit in accordance with the manufacturer’s instructions. The NanoDrop Spectrophotometer confirmed that the isolated DNA concentrations exceeded 40 ng/μL. All SNPs were genotyped using the Agena MassARRAY SNP typing on the Agena Bioscience platform, utilizing the MALDI‐TOF primer extension assay. The primers for the polymerase chain reaction (PCR) were designed using the Primer‐BLAST tool of the NCBI based on the sequence of the forward strands from the NCBI human reference genome GRCh38 (hg38). After PCR amplification of DNA samples, unincorporated dNTPs were neutralized, followed by extension. Following application of the amplified DNA to the SpectroChip arrays, genotyping was performed using a mass spectrometer equipped with TYPE 4.0 software. Using single‐base primer extension with mass‐modified terminators, the iPlex genotyping assay was utilized in the MassARRAY system to boost plexing flexibility and efficiency. The genotyping results were confirmed by two different researchers [25].

### 2.5. Statistical Analyses

The chi‐square goodness‐of‐fit test was used to evaluate the Hardy–Weinberg equilibrium (HWE) and analyze the genotype frequency distribution. The software called the family‐based association test (FBAT) was used to assess Mendelian discrepancies. Mendelian error‐displaying genotypes were set to zero. Haploview 4.2 was utilized to compute the pairwise linkage between two SNPs. SNP pairs exhibiting a D′ value more than 0.7 were deemed to be in a state of strong linkage disequilibrium (LD).

Association analyses were conducted using FBAT v1.7.2 software [26] to study the relationship between SNPs and traits. Three different inheritance models were examined: additive, dominant, and recessive. In the additive model, the log‐odds of having a trait rise linearly with the number of copies of a risk allele (two alleles > one allele > zero allele). The dominant model suggests that possessing one or two copies of an allele is equally likely to result in the trait. The recessive model indicates that two copies of an allele are required to increase the probability of having the trait. Due to the uncertain inheritance modes of autism, all three models were tested. To account for multiple testing and reduce the chance of Type I error, the permutation tests were employed. The significance level was set at *p* < 0.0042 (*α*/*n* = 0.05/12 = 0.0042), where *α* represents the desired overall significance level and *n* is the number of tests conducted. This correction helps control the increased risk of false‐positive results when performing multiple statistical tests.

For conducting many types of transmission disequilibrium tests (TDTs), including haplotype studies, the FBAT program uses generalized score statistics. In the haplotype‐based association test (HBAT), individual haplotype tests were carried out in the “biallelic” mode, whereas the global haplotype tests of association are carried out in the “multiallelic” mode. For multiple testing corrections of haplotype analyses (*n* = 10,000), permutation tests were employed. The power of this association analysis is computed using the Quanto software version 1.2.4. A population risk of 0.006 and a relative risk of 1.5 are assumed in the power calculation.

### 2.6. *In Silico* Analyses of *TRIO* Polymorphisms

A range of web‐based instruments was employed to examine the annotations of noncoding variations. The Genomics‐Tissue Expression (GTEx) database (http://www.gtexportal.org/) provided expression quantitative trait loci (eQTL) information to investigate the relationship between gene expression and genetic variation in various human tissues. HaploReg v3 (http://compbio.mit.edu/HaploReg) was applied to discern chromatin states, conservation, and regulatory motif changes in risk variants and SNPs [27]. Furthermore, VarNote (https://mulinlab.org/varnote/application.html#REG) and rVarBase (http://rv.psych.ac.cn/) were employed to determine whether the linked SNPs resided in transcription factor (TF) binding regions [28]. The Human Brain Transcriptome database (http://hbatlas.org/pages/hbtd) assessed the dynamic *TRIO* expression across various developmental and adulthood stages in regions such as the cerebellar cortex, mediodorsal nucleus of the thalamus, striatum, amygdala, hippocampus, and 11 neocortical areas [29].

## 3. Results

We analyzed 12 tag SNPs, which have captured on average 75% of the common variation with a MAF > 5%. With Agena MassARRAY SNP typing, the regenotyped samples had a genotype concordance rate of more than 99%. After 239 families underwent successful genotyping, all 12 of the chosen SNPs in *TRIO* were discovered to be polymorphic. The HWE was supported by the genotype distributions of the SNPs in children diagnosed with autism in 239 trios. Genotype frequencies and HWE data in the 239 trios are shown in Table S1.

The risk allele frequencies of the 12 SNPs in 239 trios varied from 0.046 to 0.959, and the range of these risk alleles’ identification power was 37%–87%. In Table S2, we have presented the frequencies of transmission and nontransmission of each allele to autistic children. However, the MAF values of rs4702023 calculated from our own study data did not reach 0.05, so we removed the SNP rs4702023 from the list. Allele G of rs33005 showed a substantial preferred transmission from parents to autistic children in 239 trios, according to univariate FBAT conducted under an additive model (G > T, *p* = 0.0018; Table 2). Additionally, there was a substantial preferential transmission for both the C allele of rs27479 and the A allele of rs32593 (rs27479: C > A, *p* = 0.0072; rs32593: A > G, *p* = 0.0003; Table 2). In a recessive model, the G allele of rs33005, the A allele of rs32593, and the C allele of rs27479 were also found to have a preferential transmission (rs33005: G > T, *p* = 0.0083; rs32593: A > G, *p* = 0.0034; rs27479: C > A, *p* = 0.0025; Table S3). The Z‐scores for the alleles in the dominant model were flipped and in the opposite direction (Table S4).

**Table 2:** Results of association analyses between 12 SNPs in *TRIO* and autism in 239 trios by FBAT under an additive model.

Markers	Allele	Afreq	Families	S	E(s)	Var(s)	Z	*p*	*p* ^*a*^
**rs32593**	A	0.563	180	227	199.5	58.75	3.588	0.00033	0.0064
	G	0.437	180	133	160.5	58.75	−3.588	0.00033	0.0064
**rs33005**	G	0.499	178	211	181.5	60.75	3.785	0.00015	0.0027
	T	0.501	178	145	174.5	60.75	−3.785	0.00015	0.0027
rs2440982	T	0.456	173	186	164.0	58.00	2.889	0.00387	0.0731
	C	0.544	173	160	182.0	58.00	−2.889	0.00387	0.0731
rs42551	T	0.390	159	152	134.5	55.25	2.354	0.01856	0.3272
	A	0.610	159	166	183.5	55.25	−2.354	0.01856	0.3272
rs181927	G	0.456	170	179	162.0	57.50	2.242	0.02497	0.4157
	T	0.544	170	161	178.0	57.50	−2.242	0.02497	0.4157
rs730184	A	0.103	81	50	44.0	21.50	1.294	0.19567	0.9962
	G	0.897	81	112	118.0	21.50	−1.294	0.19567	0.9962
rs30770	T	0.282	144	109	106.0	45.00	0.447	0.65472	1.0000
	G	0.718	144	179	182.0	45.00	−0.447	0.65472	1.0000
rs30773	A	0.064	53	31	28.5	14.25	0.662	0.50780	1.0000
	G	0.936	53	75	77.5	14.25	−0.662	0.50780	1.0000
rs27108	T	0.848	112	153	160.5	31.25	−1.342	0.17971	0.9921
	C	0.152	112	71	63.5	31.25	1.342	0.17971	0.9921
rs26182	T	0.853	106	144	153.5	29.25	−1.757	0.07899	0.8268
	G	0.147	106	68	58.5	29.25	1.757	0.07899	0.8268
**rs27479**	C	0.878	99	160	142.0	27.50	3.432	0.00060	0.0124
	A	0.122	99	38	56.0	27.50	−3.432	0.00060	0.0124

*Note:* Afreq, allele frequency; Fam, number of informative families; S, test statistics for the observed number of transmitted alleles; E(S), expected value of S under the null hypothesis (i.e., no linkage and no association). Bold values indicate statistically significant differences (*p* < 0.0042) after Bonferroni correction.

^a^Bonferroni‐corrected statistical results.

Subsequently, we increased the sample size to 427 trios to conduct a more in‐depth examination of the relationship between three SNPs (rs32593, rs33005, and rs27479) and autism. It was discovered that the HWE was supported by the genotype distributions of SNPs in children diagnosed with autism. In particular, the power to discover the frequencies of risk alleles for rs32593, rs33005, and rs27479 improved to more than 90% when the number of samples tested was increased to 427 trios.

In the analysis using an additive model, the univariate FBAT indicated that the G allele of rs33005, the A allele of rs32593, and the C allele of rs27479 exhibited a preferential transmission from parents to children affected with autism in 427 trios (rs33005: G > T, *p* = 0.0029; rs32593: A > G, *p* = 0.0093; rs27479: C > A, *p* = 0.0013; Table 3). Furthermore, the A allele of rs32593 demonstrated undertransmission in a recessive paradigm (rs32593: A > G, *p* = 0.1017; Table S5). Remarkably, Table S6 showed that the Z‐scores for the alleles determined by FBAT under the dominant model go in the opposite direction.

**Table 3:** Results of association analyses between three SNPs in *TRIO* in 427 trios by FBAT under an additive model.

Markers	Allele	Afreq	Fam	S	E(s)	Var(s)	Z	*p*	*p* ^*a*^
rs32593	A	0.554	313	367.00	341.00	100.00	2.600	0.0093	**0.0279**
	G	0.446	313	259.00	285.00	100.00	−2.600	0.0093	**0.0279**
rs33005	G	0.493	298	328.00	298.00	101.50	2.978	0.0029	**0.0120**
	T	0.507	298	268.00	298.00	101.50	−2.978	0.0029	**0.0120**
rs27479	C	0.880	160	249.00	227.50	44.75	3.214	0.0013	**0.0039**
	A	0.120	160	71.00	92.50	44.75	−3.214	0.0013	**0.0039**

*Note:* Afreq, allele frequency; Fam, number of informative families; S, test statistics for the observed number of transmitted alleles; E(S), expected value of S under the null hypothesis (i.e., no linkage and no association). Bold values indicate statistically significant differences (*p* < 0.0042) after Bonferroni correction.

^a^Bonferroni‐corrected statistical result.

**Figure 2:**
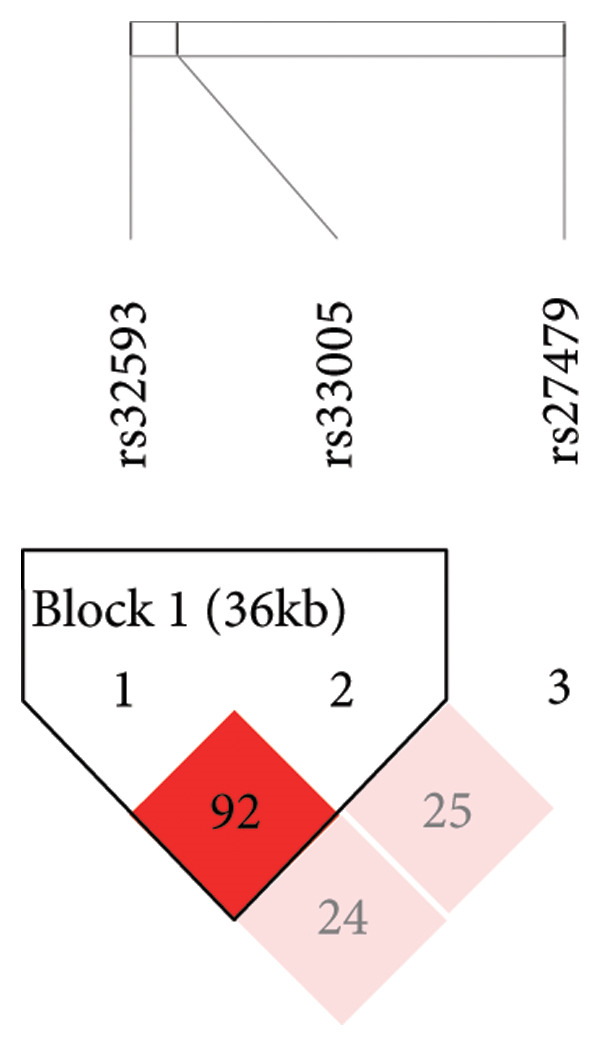
The linkage disequilibrium (LD) block of three SNPs in *TRIO* in the 427 trios. Solid spine of LD, D′ > 0.7; markers with LD (D′ < 1 and LOD > 2) are shown in red. Regions of low LD and low LOD scores (D′ < 1 and LOD < 2) are shown in pink.

An LD test revealed the presence of four LD blocks among the 12 tag SNPs in 239 trios. In particular, rs32593 and rs33005 were situated in Block 1, whereas rs27479 was located in Block 4 (Figure S1). Subsequently, haplotype analysis in 427 trios was performed exclusively for the LD block (Figure 2). It showed that one haplotype (A‐G) constructed from rs32593 and rs33005 exhibited significant excess transmission from parents to affected offspring (*p* = 0.0064; Global *p* = 0.022). The results indicated a strong association between autism and the haplotypes constructed from rs32593 and rs33005 (Table 4).

**Table 4:** Association of haplotypes constructed from rs32593 and rs33005 in *TRIO*.

Marker	Haplotypes	Freq	Fam	S	E(s)	Var(s)	Z	*p*	Global *p*	Permutation^a^ *p*
rs32593–rs33005	A‐G	0.475	273.9	320.88	292.90	105.34	2.725	0.0064	0.022	0.008
	G‐T	0.426	280.9	233.88	260.40	107.56	−2.558	0.0105		
	A‐T	0.081	107.0	61.12	65.59	28.55	−0.837	0.4027		
	G‐T	0.018	29.2	18.125	15.09	7.35	1.117	0.2639		

^a^Whole marker permutation test using chisq sum *p*‐value; the number of permutations is 10,000; freq, estimation of haplotype frequencies; Fam, number of informative families; S, test statistics for the observed number of transmitted alleles; E(S), expected value of S under the null hypothesis (i.e., no linkage and no association).

To test whether the three SNPs in *TRIO* remain positive in the association with autism in the European sample, we conducted an analysis of genome‐wide association study (GWAS) results in ASDs obtained from the Psychiatric Genomics Consortium (PGC). However, our study found that the three *TRIO* markers examined were not linked to autism in the PGC sample (Table S7), which indicated that the genetic association between *TRIO* and autism might have population specificity. Although no exact matches for our positive SNPs were identified in the PGC ASD GWAS dataset, we further analyzed PGC SNPs in high LD (*r* ^2^ > 0.8) with our positive SNPs. For the PGC‐linked SNP corresponding to rs33005, the G allele (consistent with the risk allele identified in our study) exhibited a directionally consistent effect (OR = 1.08, *p* = 0.058), albeit without reaching statistical significance. This consistent direction supports our finding that rs33005 is associated with ASD. The lack of statistical significance, however, may be attributed to either ethnic heterogeneity (our study focused on the Han Chinese in Beijing [CHB] population, whereas the PGC dataset primarily includes European populations) or differences in sample size between the two studies (Table 5).

**Table 5:** The results of autism GWAS from the Psychiatric Genomics Consortium (PGC).

CHR	SNP	BP	A1	A2	INFO	OR	SE	*P*	Beta
5	rs33005	14206537	T	G	0.994	0.97395	0.0139	**0.058**	−0.026395311
5	rs32593	14169709	A	G	1.01	1.00642	0.014	0.6466	0.00639948
5	rs27479	14499395	A	C	0.99	1.01521	0.0204	0.4601	0.015095488

*Note:* The bold values indicate that the G allele of rs33005 in the PGC ASD GWAS dataset shows a directionally consistent effect, which supports our finding that rs33005 is associated with ASD.

CHR, chromosome; SNP, single‐nucleotide polymorphism; BP, base pair position; A1, effect allele; A2, noneffect allele; INFO, information score; OR, odds ratio; SE, standard error; *p*, *p*‐value; beta, regression coefficient.

The expression level of *TRIO* undergoes a rapid increase before postnatal Day 100 and remains consistently high in multiple brain regions throughout an individual’s life (Figure S2). To investigate the potential impact of these positively associated SNPs on *TRIO* expression in individuals with autism, we conducted an in silico analysis. The functional prediction of noncoding variants revealed that rs32593 and rs27479 might modify two motifs and one motif, respectively (Table S8). Additionally, rs32593, rs33005, and rs72479 might influence the binding of enhancers to the *TRIO* genome (Table S9). eQTL analysis also suggested that rs32593, rs33005, and rs27479 might affect *TRIO* expression in the cortex or cerebellum (Table S10). These findings suggested that these positively associated SNPs could potentially be regulatory SNPs that impact the physiological function of *TRIO* and are involved in the etiology of autism.

## 4. Discussion

In this study, we conducted a family‐based association study to explore the link between the GEF *TRIO* and autism in the Chinese Han population. Our findings revealed a significant association between autism and three common variants (rs32593, rs33005, and rs27479) within *TRIO* in 427 Han Chinese autism trios. Additionally, the haplotypes formed by rs32593 and rs33005 showed a notable association with autism. Functional analysis suggested that these three *TRIO* SNPs might influence gene expression levels by residing in regulatory regions or impacting TF binding.

Genetic studies indicated that common variations account for approximately 15%–50% of the genetic risk for ASD, and the cumulative effect of these SNPs might increase the risk of ASD [2]. Thus, common variability within SNPs could contribute to the emergence of autism, the associated features in families (the broader autism phenotype), and the increased incidence of autism in offspring of parents with increased autistic and autistic traits in the general population [2, 17, 30]. However, the relationship between common variations in *TRIO* and ASD is not fully understood. To our knowledge, we present here the first report on the association between common variations in *TRIO* and autism in the Chinese Han population. If common variations associated with autism can be identified at the genetic level, high‐risk individuals can be recognized earlier in a larger population, enabling early intervention. According to previous studies, mutations in *TRIO* affect neuronal migration and synaptic function. As the common variations in *TRIO* unfold, animal models and system biology methods will enable the identification of diverse etiologies and convergent molecular and cellular pathways crucial for neurodevelopment [17, 30]. These studies help us gain a deeper understanding of the neurobiological basis of autism. Understanding how these genetic mutations impact brain development and function can provide important clues about the pathological mechanisms of autism. Loss‐of‐function or gain‐of‐function mutations in *TRIO* are associated with the behavioral phenotype of autism. Therefore, the *TRIO*‐related signaling pathways might serve as potential therapeutic targets. For example, modulating the activity of *TRIO* through medication or other interventions might help alleviate symptoms in individuals with autism.

However, the SNPs including these three positively associated markers in *TRIO* were not linked to European ASDs in the PGC sample. The inconsistent finding might be attributed to several factors. Firstly, there could be ethnic differences in genetic heterogeneity. For instance, the MAF of rs32593 varies between CHB (Han Chinese in Beijing, China) and CEU (Utah residents with Northern and Western European ancestry from the CEPH collection) populations. Our results revealed a MAF of 0.446 for rs32593 in the CHB population, whereas it is approximately 0.398 in the CEU population. Secondly, our study employed a family‐based association approach to mitigate population stratification, which differs from previous studies predominantly based on case–control designs. Thirdly, discrepancies in clinic heterogeneity among patients, including demographic characteristics and gene–environment interactions, might contribute to the conflicting outcomes.

The cellular etiology of ASDs involves abnormalities in one or more developmental events, including neurogenesis, neuronal migration, axonal projection, dendritic development, dendritic spine formation, synaptogenesis, and synaptic remodeling. In many research studies, *TRIO* has been identified to be involved in these developmental events [31, 32]. For example, it controls the axon development of cerebellar granule cells (including growth, branching, and guidance), making it an essential molecule for cerebellar development [30]. Our previous study and Eid L et al. [32] revealed that *TRIO* is also involved in the migration of cortical interneurons (INs). The functional mutations in the *TRIO* gene are closely associated with ASD, particularly mutations in the GEF1 domain [33]. After the complete knockout of *Trio*, the number of embryonic inhibitory neurons migrating from the ganglionic eminence to the cortex is reduced, leading to severe social deficits, repetitive stereotyped behaviors, and other autism‐like behavioral disorders in mice [34]. The eQTL analysis in our study revealed potential impacts of rs32593, rs33005, and rs27479 on *TRIO* expression in the cortex and cerebellum. These findings imply that these three SNPs might serve as regulatory SNPs, pending validation through further biological experiments. Furthermore, functional predictions from online databases suggest that rs32593, rs33005, and rs27479 modulate enhancer binding to the *TRIO* genome, potentially contributing to the regulation of *TRIO* expression in the human brain.

In conclusion, our study hints at *TRIO’*s potential involvement in the pathogenesis of autism. In this study, we identified an association of rs32593, rs33005, and rs27479 in *TRIO* with autism. Functional analysis suggested that these three SNPs in *TRIO* could influence gene expression levels by residing in regulatory regions or impacting TF binding. Our findings warrant replication and validation in future studies with larger sample sizes and diverse populations. Further exploration of the functional consequences of these variations is crucial for unraveling the underlying mechanisms of autism.

## Disclosure

Han Shen and Xiaoxuan Sun are the co‐first authors.

## Conflicts of Interest

The authors declare no conflicts of interest.

## Author Contributions

Han Shen and Xiaoxuan Sun contributed equally to this work.

## Funding

The authors thank all subjects who participated in this study and our colleagues for their assistance in recruiting patients. This study was supported by grants from the Key‐Area Research and Development Program of Guangdong Province (2019B030335001), the National Natural Science Foundation of China (Grant Numbers 82471566, 82571757, 82271576 and 82571759), the China Disabled Persons’ Federation (CDPF2023KF00001) and the Nonprofit Central Research Institute Fund of Chinese Academy of Medical Sciences (2023‐PT320‐08).

## Supporting Information

Additional supporting information can be found online in the Supporting Information section.

## Supporting information


**Supporting Information 1** Figure S1: The linkage disequilibrium (LD) block of 12 SNPs in *TRIO* in the 239 trios.

**Supporting Information 2** Figure S2: Dynamic expression levels of *TRIO* in the human brain throughout life.

**Supporting Information 3** Table S1: Information on the selected 12 SNPs in *TRIO* and genotype frequencies in 239 autism trios.

**Supporting Information 4** Table S2: Allele transmitted/untransmitted times of 12 SNPs in *TRIO* in 427 autism trios.

**Supporting Information 5** Table S3: Results of association analyses between 12 SNPs in *TRIO* and autism in 239 trios by FBAT under a recessive model.

**Supporting Information 6** Table S4: Results of association analyses between 12 SNPs in *TRIO* and autism in 239 trios by FBAT under a dominant model.

**Supporting Information 7** Table S5: Association results between three SNPs in *TRIO* and autism in 427 trios by FBAT under a recessive model.

**Supporting Information 8** Table S6: Association results between three SNPs in *TRIO* and autism in 427 trios by FBAT under a dominant model.

**Supporting Information 9** Table S7: The results of autism GWAS from the Psychiatric Genomics Consortium (PGC).

**Supporting Information 10** Table S8: The expression quantitative trait loci (eQTL) analyses of three SNPs in *TRIO*.

**Supporting Information 11** Table S9: Functional annotation for rs27479 and rs32593.

**Supporting Information 12** Table S10: Results of VarNote in predicting the effect of rs32593, rs33005, and rs27479 on transcription factors.

## Data Availability

All relevant data are within the paper and its Supporting Information files.
