# Supplementary material for: Common Genetic Variants in TRIO Are Associated With Autism in Chinese Han Population
Source: Genet Res (Camb). 2025 Dec 17;2025:7762302. doi: 10.1155/genr/7762302 (PMC12721762; doi:10.1155/genr/7762302)
Supplement: Supplementary file 1 — Supporting Information 1 Figure S1: The linkage disequilibrium (LD) block of 12 SNPs in TRIO in the 239 trios. [file GENR-2025-7762302-s002.docx]

**Figure S1. The linkage disequilibrim (LD) block of 12 SNPs in *TRIO* in the 239 trios in terms of both D' and r² values.**

D'：


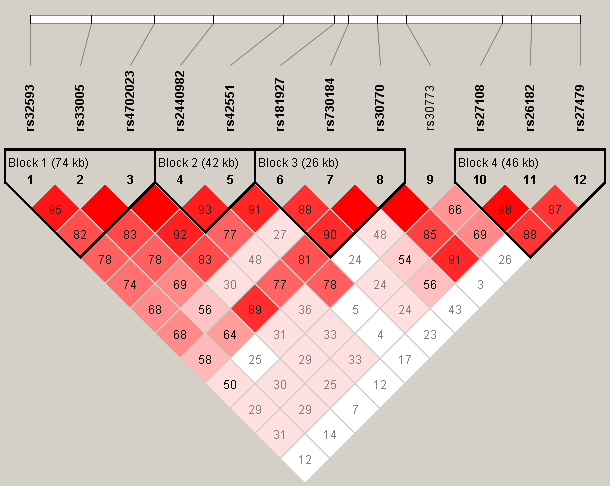


R-squared：


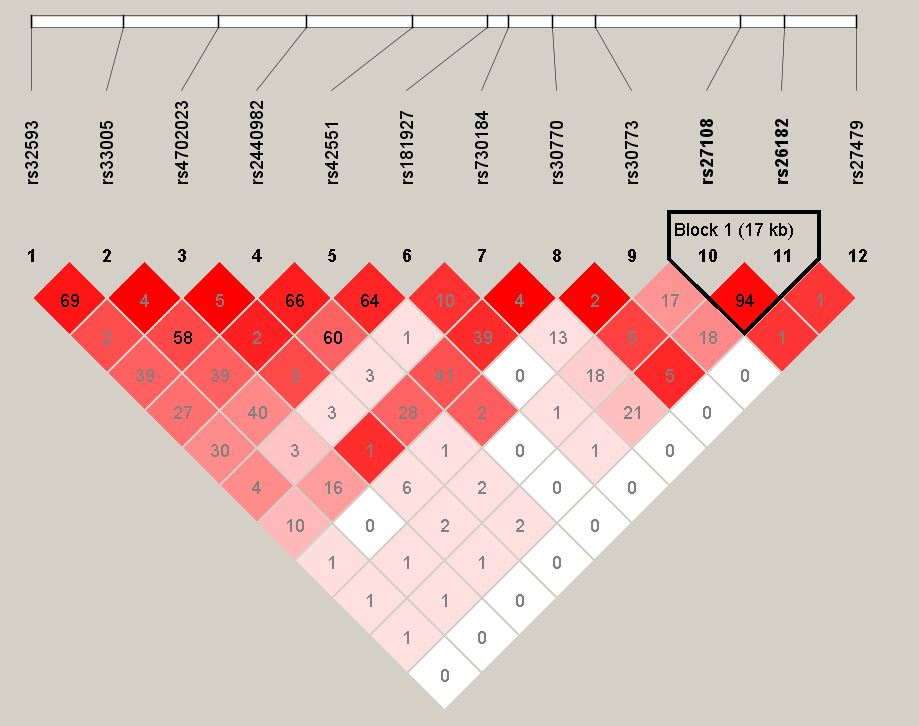


Solid spine of LD, D’>0.7; Markers with LD (D’<1 and LOD>2) are shown in red. Regions of low LD and low LOD scores (D’<1 and LOD<2) are shown in pink.
