# Supplementary material for: Common Genetic Variants in TRIO Are Associated With Autism in Chinese Han Population
Source: Genet Res (Camb). 2025 Dec 17;2025:7762302. doi: 10.1155/genr/7762302 (PMC12721762; doi:10.1155/genr/7762302)
Supplement: Supplementary file 2 — Supporting Information 2 Figure S2: Dynamic expression levels of TRIO in the human brain throughout life. [file GENR-2025-7762302-s001.docx]

**Figure S2. Dynamic expression levels of *TRIO* in the human brain throughout life.**


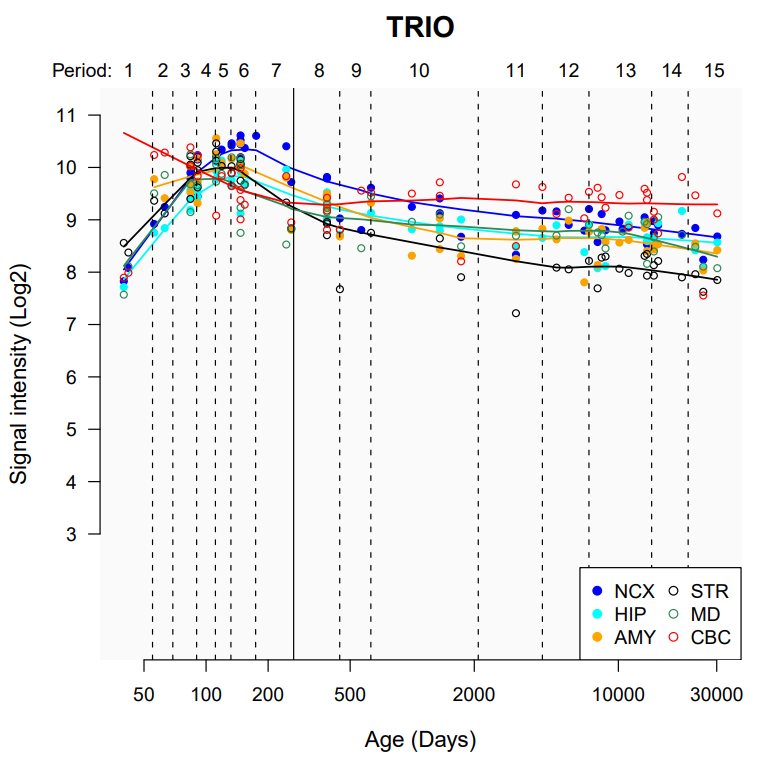


NCX, 11 areas of neocortex; STR, striatum; HIP, hippocampus; CBC, the cerebellar cortex; MD, mediodorsal nucleus of the thalamus; AMY, amygdala
