# Supplementary material for: Common Genetic Variants in TRIO Are Associated With Autism in Chinese Han Population
Source: Genet Res (Camb). 2025 Dec 17;2025:7762302. doi: 10.1155/genr/7762302 (PMC12721762; doi:10.1155/genr/7762302)
Supplement: Supplementary file 3 — Supporting Information 3 Table S1: Information on the selected 12 SNPs in TRIO and genotype frequencies in 239 autism trios. [file GENR-2025-7762302-s011.docx]

**Table S1.** **Information of the selected 12 SNPs in *TRIO* and genotype frequencies in 239 autism trios**

| **Marker** | **Chr. position** | **Genotype frequencies in children** | | | ***p* _HWE_ ^a^** | **Genotype frequencies in parents** | | | ***p* _HWE_ ^b^** |
| --- | --- | --- | --- | --- | --- | --- | --- | --- | --- |
| rs32593 | 14222709 | AA | AG | GG | 0.993 | AA | AG | GG | 0.759 |
|  |  | 92 | 112 | 34 |  | 149 | 237 | 89 |  |
| rs33005 | 14259537 | GG | GT | TT | 0.620 | GG | GT | TT | 0.521 |
|  |  | 73 | 121 | 44 |  | 115 | 245 | 116 |  |
| rs4702023 | 14297357 | GG | GA | AA | 0.435 | GG | GA | AA | 0.820 |
|  |  | 216 | 23 | 0 |  | 435 | 37 | 1 |  |
| rs2440982 | 14332540 | TT | TC | CC | 0.516 | TT | TC | CC | 0.820 |
|  |  | 58 | 124 | 56 |  | 99 | 233 | 143 |  |
| rs42551 | 14374785 | TT | TA | AA | 0.813 | TT | TA | AA | 0.581 |
|  |  | 44 | 115 | 80 |  | 75 | 221 | 181 |  |
| rs181927 | 14404946 | GG | GT | TT | 0.644 | GG | GT | TT | 0.648 |
|  |  | 55 | 122 | 60 |  | 94 | 236 | 136 |  |
| rs730184 | 14413436 | AA | AG | GG | 0.044 | AA | AG | GG | 0.592 |
|  |  | 0 | 55 | 184 |  | 6 | 85 | 386 |  |
| rs30770 | 14431068 | TT | TG | GG | 0.512 | TT | TG | GG | 0.110 |
|  |  | 22 | 94 | 123 |  | 45 | 179 | 253 |  |
| rs30773 | 14448220 | AA | AG | GG | 0.252 | AA | AG | GG | 0.972 |
|  |  | 0 | 33 | 206 |  | 2 | 57 | 417 |  |
| rs27108 | 14505841 | TT | TC | CC | 0.747 | TT | TC | CC | 0.757 |
|  |  | 165 | 68 | 6 |  | 343 | 124 | 10 |  |
| rs26182 | 14523509 | TT | TG | GG | 0.795 | TT | TG | GG | 0.760 |
|  |  | 165 | 67 | 6 |  | 347 | 117 | 11 |  |
| rs27479 | 14552395 | CC | CA | AA | 0.147 | CC | CA | AA | 0.081 |
|  |  | 198 | 41 | 0 |  | 363 | 110 | 3 |  |

^a^ Hardy-Weinberg equilibrium *p* value for genotype distributions in children affected with autism; ^b^ Hardy-Weinberg equilibrium *p* value for genotype distributions in parents.
