# Supplementary material for: Common Genetic Variants in TRIO Are Associated With Autism in Chinese Han Population
Source: Genet Res (Camb). 2025 Dec 17;2025:7762302. doi: 10.1155/genr/7762302 (PMC12721762; doi:10.1155/genr/7762302)
Supplement: Supplementary file 4 — Supporting Information 4 Table S2: Allele transmitted/untransmitted times of 12 SNPs in TRIO in 427 autism trios. [file GENR-2025-7762302-s010.docx]

**Table S2.** **Allele transmitted/untransmitted times of 12 SNPs in *TRIO* in 427 autism trios**

| **Marker** | **Allele** | **Transmitted times** | **Untransmitted times** |
| --- | --- | --- | --- |
| rs32593 | A | 296 | 239 |
|  | G | 180 | 235 |
| rs33005 | G | 267 | 208 |
|  | T | 209 | 268 |
| rs4702023 | G | 455 | 452 |
|  | A | 23 | 16 |
| rs2440982 | T | 240 | 191 |
|  | C | 236 | 283 |
| rs42551 | T | 203 | 168 |
|  | A | 275 | 308 |
| rs181927 | G | 232 | 192 |
|  | T | 242 | 266 |
| rs730184 | A | 55 | 42 |
|  | G | 423 | 434 |
| rs30770 | T | 138 | 131 |
|  | G | 340 | 345 |
| rs30773 | A | 33 | 28 |
|  | G | 445 | 446 |
| rs27108 | T | 398 | 412 |
|  | C | 80 | 64 |
| rs26182 | T | 397 | 414 |
|  | G | 79 | 60 |
| rs27479 | C | 437 | 399 |
|  | A | 41 | 75 |
