# Supplementary material for: Common Genetic Variants in TRIO Are Associated With Autism in Chinese Han Population
Source: Genet Res (Camb). 2025 Dec 17;2025:7762302. doi: 10.1155/genr/7762302 (PMC12721762; doi:10.1155/genr/7762302)
Supplement: Supplementary file 5 — Supporting Information 5 Table S3: Results of association analyses between 12 SNPs in TRIO and autism in 239 trios by FBAT under a recessive model. [file GENR-2025-7762302-s009.docx]

**Table S3. Results of association analyses between 12 SNPs in *TRIO* and autism in 239 trios by FBAT under a recessive model**

| **Marker** | **Allele** | **Afreq** | **Fam** | **S** | **E (S)** | **Var (S)** | **Z** | ***p*** |
| --- | --- | --- | --- | --- | --- | --- | --- | --- |
| **rs32593** | A | 0.563 | 137 | 71.0 | 54.75 | 30.81 | 2.927 | **0.0034** |
|  | G | 0.437 | 98 | 24.0 | 35.25 | 26.06 | -2.451 | **0.0142** |
| **rs33005** | G | 0.499 | 125 | 60.0 | 46.25 | 27.18 | 2.637 | **0.0083** |
|  | T | 0.501 | 118 | 27.0 | 42.75 | 25.43 | -3.123 | **0.0017** |
| rs4702023 | G | 0.959 | 37 | 16.0 | 18.50 | 9.25 | -0.822 | 0.4110 |
|  | A | 0.041 | 0 | - | - | - | - | - |
| **rs2440982** | T | 0.456 | 107 | 48.0 | 38.75 | 23.06 | 1.926 | **0.0540** |
|  | C | 0.544 | 125 | 35.0 | 47.75 | 27.56 | -2.429 | **0.0151** |
| rs42551 | T | 0.390 | 86 | 36.0 | 27.50 | 17.62 | 2.025 | 0.0429 |
|  | A | 0.610 | 135 | 43.0 | 52.00 | 29.87 | -1.647 | 0.0996 |
| rs181927 | G | 0.456 | 107 | 43.0 | 38.50 | 23.00 | 0.938 | 0.3480 |
|  | T | 0.544 | 123 | 34.0 | 46.50 | 27.00 | -2.406 | 0.0161 |
| rs730184 | A | 0.103 | 6 | - | - | - | - | - |
|  | G | 0.897 | 80 | 31.0 | 38.75 | 19.68 | -1.747 | 0.0806 |
| rs30770 | T | 0.282 | 52 | 20.0 | 17.00 | 10.75 | 0.915 | 0.3601 |
|  | G | 0.718 | 128 | 55.0 | 55.00 | 29.75 | 0.000 | 1.0000 |
| rs30773 | A | 0.064 | 4 | - | - | - | - | - |
|  | G | 0.936 | 53 | 22.0 | 25.50 | 13.00 | -0.971 | 0.3316 |
| rs27108 | T | 0.848 | 111 | 47.0 | 52.25 | 26.93 | -1.012 | 0.3117 |
|  | C | 0.152 | 14 | 6.0 | 3.75 | 2.68 | 1.372 | 0.1699 |
| rs26182 | T | 0.853 | 106 | 44.0 | 50.25 | 25.81 | -1.230 | 0.2186 |
|  | G | 0.147 | 11 | 6.0 | 2.75 | 2.06 | 2.263 | 0.0236 |
| **rs27479** | C | 0.878 | 98 | 61.0 | 46.25 | 23.81 | 3.023 | **0.0025** |
|  | A | 0.122 | 12 | 0.0 | 3.25 | 2.31 | -2.137 | **0.0325** |

Afreq, allele frequency; Fam, number of informative families; S, test statistics for the observed number of transmitted alleles; E(S), expected value of S under the null hypothesis (i.e., no linkage and no association).
