# Supplementary material for: Common Genetic Variants in TRIO Are Associated With Autism in Chinese Han Population
Source: Genet Res (Camb). 2025 Dec 17;2025:7762302. doi: 10.1155/genr/7762302 (PMC12721762; doi:10.1155/genr/7762302)
Supplement: Supplementary file 6 — Supporting Information 6 Table S4: Results of association analyses between 12 SNPs in TRIO and autism in 239 trios by FBAT under a dominant model. [file GENR-2025-7762302-s008.docx]

**Table S4. Results of association analyses between 12 SNPs in *TRIO* and autism in 239 trios by FBAT under a dominant model**

| **Marker** | **Allele** | **Afreq** | **Fam** | **S** | **E (S)** | **Var (S)** | **Z** | ***p*** |
| --- | --- | --- | --- | --- | --- | --- | --- | --- |
| **rs32593** | A | 0.563 | 98 | 74.0 | 62.75 | 21.06 | 2.451 | **0.0142** |
|  | G | 0.437 | 137 | 66.0 | 82.25 | 30.81 | -2.927 | **0.0034** |
| **rs33005** | G | 0.499 | 118 | 91.0 | 75.25 | 25.43 | 3.123 | **0.0017** |
|  | T | 0.501 | 125 | 65.0 | 78.75 | 27.18 | -2.637 | **0.0083** |
| rs4702023 | G | 0.959 | 0 | - | - | - | - | - |
|  | A | 0.041 | 37 | 21.0 | 18.50 | 9.25 | 0.822 | 0.4110 |
| rs2440982 | T | 0.456 | 125 | 90.0 | 77.25 | 27.56 | 2.429 | 0.0151 |
|  | C | 0.544 | 107 | 59.0 | 68.25 | 23.06 | -1.926 | 0.0540 |
| rs42551 | T | 0.390 | 135 | 92.0 | 83.00 | 29.87 | 1.647 | 0.0996 |
|  | A | 0.610 | 86 | 50.0 | 58.50 | 17.62 | -2.025 | 0.0429 |
| rs181927 | G | 0.456 | 123 | 89.0 | 76.50 | 27.00 | 2.406 | 0.0161 |
|  | T | 0.544 | 107 | 64.0 | 68.50 | 23.00 | -0.938 | 0.3480 |
| rs730184 | A | 0.103 | 80 | 49.0 | 41.25 | 19.68 | 1.747 | 0.0806 |
|  | G | 0.897 | 6 | - | - | - | - | - |
| rs30770 | T | 0.282 | 128 | 73.0 | 73.00 | 29.75 | 0.000 | 1.0000 |
|  | G | 0.718 | 52 | 32.0 | 35.00 | 10.75 | -0.915 | 0.3601 |
| rs30773 | A | 0.064 | 53 | 31.0 | 27.50 | 13.00 | 0.971 | 0.3316 |
|  | G | 0.936 | 4 | - | - | - | - | - |
| rs27108 | T | 0.848 | 14 | 8.0 | 10.25 | 2.68 | -1.372 | 0.1699 |
|  | C | 0.152 | 111 | 64.0 | 58.75 | 26.93 | 1.012 | 0.3117 |
| rs26182 | T | 0.853 | 11 | 5.0 | 8.25 | 2.06 | -2.263 | 0.0236 |
|  | G | 0.147 | 106 | 62.0 | 55.75 | 25.81 | 1.230 | 0.2186 |
| **rs27479** | C | 0.878 | 12 | 12.0 | 8.75 | 2.31 | 2.137 | **0.0325** |
|  | A | 0.122 | 98 | 37.0 | 51.75 | 23.81 | -3.023 | **0.0025** |

Afreq, allele frequency; Fam, number of informative families; S, test statistics for the observed number of transmitted alleles; E(S), expected value of S under the null hypothesis (i.e., no linkage and no association).
