# Supplementary material for: Common Genetic Variants in TRIO Are Associated With Autism in Chinese Han Population
Source: Genet Res (Camb). 2025 Dec 17;2025:7762302. doi: 10.1155/genr/7762302 (PMC12721762; doi:10.1155/genr/7762302)
Supplement: Supplementary file 7 — Supporting Information 7 Table S5: Association results between three SNPs in TRIO and autism in 427 trios by FBAT under a recessive model. [file GENR-2025-7762302-s007.docx]

**Table S5. Association results between 3 SNPs in *TRIO* and autism in 427 trios by FBAT under a recessive model**

| **Markers** | **Allele** | **Afreq** | **Fam** | **S** | **E(s)** | **Var(s)** | **Z** | ***p*** |
| --- | --- | --- | --- | --- | --- | --- | --- | --- |
| **rs32593** | A | 0.554 | 228 | 104.00 | 92.25 | 51.56 | 1.636 | 0.1017 |
|  | G | 0.446 | 172 | 50.00 | 64.25 | 37.56 | -2.325 | 0.0200 |
| **rs33005** | G | 0.493 | 203 | 87.00 | 74.50 | 44.00 | 1.884 | 0.0595 |
|  | T | 0.507 | 203 | 57.00 | 74.50 | 44.00 | -2.638 | 0.0083 |
| **rs27479** | C | 0.880 | 157 | 92.00 | 73.75 | 38.06 | 2.958 | 0.0030 |
|  | A | 0.120 | 22 | 3.00 | 6.25 | 4.31 | -1.565 | 0.1175 |

Afreq, allele frequency; Fam, number of informative families; S, test statistics for the observed number of transmitted alleles; E(S), expected value of S under the null hypothesis (i.e., no linkage and no association).
