# Supplementary material for: Common Genetic Variants in TRIO Are Associated With Autism in Chinese Han Population
Source: Genet Res (Camb). 2025 Dec 17;2025:7762302. doi: 10.1155/genr/7762302 (PMC12721762; doi:10.1155/genr/7762302)
Supplement: Supplementary file 10 — Supporting Information 10 Table S8: The expression quantitative trait loci (eQTL) analyses of three SNPs in TRIO. [file GENR-2025-7762302-s004.docx]

**Table S8.** **The expression quantitative trait loci analyses (eQTL) of 3 SNPs in *TRIO***

| **geneSymbol** | **marker** | **rsid** | **exprID** | **chr** | **start** | **stop** | **aveALL** | **CRBL** | **FCTX** | **HIPP** | **OCTX** | **PUTM** | **TCTX** |
| --- | --- | --- | --- | --- | --- | --- | --- | --- | --- | --- | --- | --- | --- |
| TRIO | chr5:14499395 | rs27479 | 2802506 | chr5 | 14143590 | 14532215 | 3.2-01 | 5.0e-01 | 2.3e-03 | 9.2e-01 | 3.3e-01 | 4.1e-01 | 3.9e-01 |
| TRIO | chr5:14169709 | rs32593 | 2802620 | chr5 | 14143590 | 14532215 | 4.3e-01 | 6.2e-01 | 6.1e-03 | 2.4e-01 | 1.1e-01 | 5.5e-01 | 8.9e-01 |
| TRIO | chr5:14206537 | rs33005 | 2802400 | chr5 | 14143590 | 14532215 | 4.3e-01 | 6.7e-03 | 9.8e-01 | 8.6e-01 | 8.4e-01 | 8.3e-01 | 3.1e-01 |
| TRIO | chr5:14206537 | rs33005 | 2802507 | chr5 | 14143590 | 14532215 | 9.5e-01 | 3 2e-01 | 1.6e-01 | 3.7e-01 | 2.3e-01 | 6.8e-02 | 7.2e-03 |

CRBL: Cerebellum; FCTX: Prefrontal Cortex; HIPP: Hippocampus; OCTX: Orbitofrontal Cortex; PUTM:Posterior Medial Pulvinar Nucleus; TCTX: Temporal Cortex
